# Sustained cross‐presentation capacity of murine splenic dendritic cell subsets in vivo

**DOI:** 10.1002/eji.201747372

**Published:** 2018-05-17

**Authors:** Nataschja I. Ho, Marcel G. M. Camps, Edwin F. E. de Haas, Ferry Ossendorp

**Affiliations:** ^1^ Department of Immunohematology and Blood Transfusion Leiden University Medical Center Leiden The Netherlands

**Keywords:** Antigen presentation/processing, CD4 T cells, CD8 T cells, Cross presentation/priming, Dendritic cells

## Abstract

An exclusive feature of dendritic cells (DCs) is their ability to cross‐present exogenous antigens in MHC class I molecules. We analyzed the fate of protein antigen in antigen presenting cell (APC) subsets after uptake of naturally formed antigen‐antibody complexes in vivo. We observed that murine splenic DC subsets were able to present antigen in vivo for at least a week. After ex vivo isolation of four APC subsets, the presence of antigen in the storage compartments was visualized by confocal microscopy. Although all APC subsets stored antigen for many days, their ability and kinetics in antigen presentation was remarkably different. CD8α^+^ DCs showed sustained MHC class I‐peptide specific CD8^+^ T‐cell activation for more than 4 days. CD8α^−^ DCs also presented antigenic peptides in MHC class I but presentation decreased after 48 h. In contrast, only the CD8α^−^ DCs were able to present antigen in MHC class II to specific CD4^+^ T cells. Plasmacytoid DCs and macrophages were unable to activate any of the two T‐cell types despite detectable antigen uptake. These results indicate that naturally occurring DC subsets have functional antigen storage capacity for prolonged T‐cell activation and have distinct roles in antigen presentation to specific T cells in vivo.

## Introduction

Dendritic cells (DCs) are professional antigen presenting cells (APCs) that can capture, process and present exogenous antigen on MHC class I (MHCI) molecules. This cross‐presentation pathway is considered to be a specialized function of DCs. We have previously demonstrated that the uptake of the protein antigen ovalbumin (OVA) bound to OVA‐specific IgG antibodies, also called OVA immune complexes (OVA IC), by DCs is at least 100‐fold more efficient than uptake of free OVA (1). This antibody‐dependent uptake route of OVA IC results in DC maturation, specific CD8^+^ T‐cell priming and tumor protection in vivo 2, 3. Moreover, we have reported that DCs can store OVA IC for many days in a lysosome‐like organelle, distinct from MHC class II compartments or MHCI processing/ loading compartments. Despite the rapid turnover rate of MHCI‐peptide complexes on the cell surface, this storage compartment serves as an antigen source for continuous supply of MHCI ligands and thereby enhancing sustained cross‐presentation to CD8^+^ T cells 1.

Lymphoid organs harbor different DC subsets in vivo and their role in antigen presentation have been studied extensively. The secondary lymphoid organs in mouse contain two main DC subtypes: plasmacytoid DCs (pDCs) and conventional DCs (cDCs). In murine spleen, resident cDCs can be further subdivided into CD8α^+^ DCs and CD8α^−^ DCs based on the expression of a wide variety of surface markers 4, 5. Although both cDC subsets have the ability to take up and present exogenous antigens on MHCI, CD8α^+^ DCs are known for their specialized and much more efficient cross‐presentation of cell‐associated, antibody‐bound, or soluble antigen to CD8^+^ T cells in vivo and ex vivo 6, 7, 8, 9, 10, 11. On the other hand, CD8α^−^ DCs preferentially present antigen on MHCII to CD4^+^ T cells to a greater extent than CD8α^+^ DCs.

In the current study we injected mice sequentially with anti‐OVA IgG and OVA to form OVA IC in vivo. We have previously demonstrated that this natural formation of antigen‐IgG complexes in vivo leads to efficient antigen cross‐presentation to CD8^+^ T cells in which C1q plays an important role 12, 13. Four APC subsets from murine spleen were studied here: CD8α^+^ DCs, CD8α^−^ DCs, pDCs and macrophages. We show that all APC subsets were able to take up and store antigen for several days in vivo. However, their ability and kinetics in antigen presentation was remarkably different. CD8α^+^ DCs showed more efficient MHCI cross‐presentation to CD8^+^ T cells for several days compared to CD8α^−^ DCs. Only CD8α^−^ DCs showed effective and prolonged MHCII presentation to CD4^+^ T cells. Despite detectable uptake of antigen, pDCs and macrophages were incapable to activate T cells. Our results show for the first time that naturally occurring DC subsets have functional compartments to store antigen for prolonged T‐cell activation in vivo and each DC subset appears to play a distinct role in their antigen presentation function to specific T cells.

## Results

### Long lasting MHCI and MHCII antigen presentation in vivo

**Figure 1:**
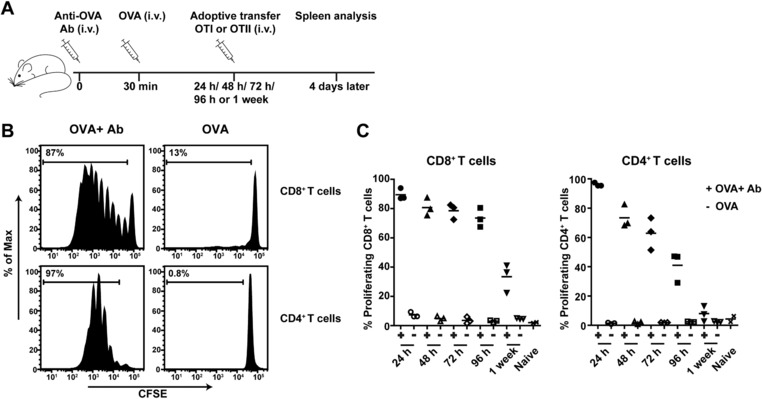
Sustained T‐cell proliferation in vivo. C57BL/6 (BL/6) mice were i.v. injected with anti‐OVA IgG (Ab) followed by OVA i.v. injection 30 min later. At different time points mice were i.v. injected with CFSE‐labeled spleen cells from OTI or OTII mice. Spleens were then collected after 4 days and analyzed by flow cytometry (A). OTI (CD8^+^/ CD90.1^+^ cells) and OTII (CD4^+^/ CD45.1^+^ cells) proliferation analysis (percentages indicated) from the spleens of mice that received OVA and Ab or OVA only injections for 24 h (B), and percentage proliferated T cells of three mice per group indicated for all time points (24 h/48 h/72 h/96 h/1 week) combined (C). Flow cytometry data are from a single experiment with three mice per group, representative of two experiments.

We previously reported that DCs have the ability to conserve exogenous protein antigen for several days in intracellular antigen containing compartments which facilitate prolonged antigen cross‐presentation to CD8^+^ T cells 1. This specialized function of DCs has primarily been shown using cultured dendritic cells in vitro. To analyze the capacity and duration of naturally occurring APC subsets to present antigen to CD8^+^ and CD4^+^ T cells in vivo, naïve C57BL/6 mice were first intravenously injected with OVA‐specific IgG antibody and after a recovery period of 30 min followed by an i.v. injection of OVA protein antigen (Fig. 1A). Separate injection of antibody followed by antigen will allow natural formation of antigen‐antibody immune complexes (IC). Antigen presentation in vivo was analyzed by injecting OVA‐specific transgenic CD8^+^ and CD4^+^ T cells, OTI or OTII cells respectively, several time points after OVA antigen administration. Twenty four hours after IgG and OVA injection strong CD8^+^ and CD4^+^ T‐cell proliferation was observed (Fig. 1B). Mice injected with only OVA did not activate either of the T‐cell subsets, underlining the efficiency of antibody‐mediated targeting of soluble protein. Mice that received OTI cells 24 h after IgG and OVA injection showed ∼90% T‐cell proliferation. This effect hardly diminished in time to ∼80% after 4 days and even one week after IgG and OVA injection 30–40% of the CD8^+^ T cells still proliferated (Fig. 1C). The same we observed for OTII CD4^+^ T‐cell proliferation. Although the proliferation of CD4^+^ T cells decreased faster in time compared to CD8^+^ T cells, there was still some activity detected after 1 week. These results show that in vivo formed antigen‐antibody complexes are efficiently presented to CD8^+^ and CD4^+^ T cells for at least a week suggesting that antigen is stored in APCs for many days.

### Effective and sustained cytotoxic T‐cell killing capacity

**Figure 2:**
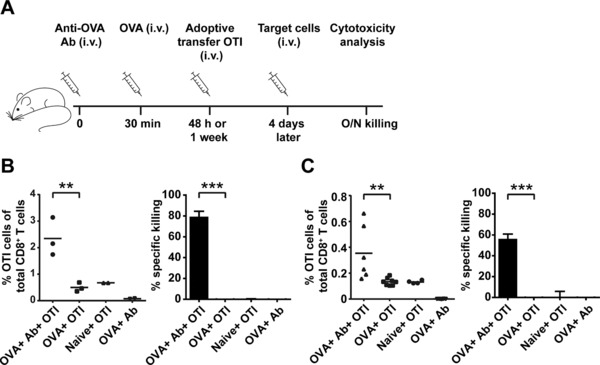
Sustained induction of cytotoxic T‐cell activity in vivo. BL/6 mice were injected i.v. with Ab followed 30 min later by OVA i.v. injection (A). After 48 h (B) or 1 week (C), mice received i.v. adoptive transfer of OTI cells, followed by target cells 4 days later. OTI cell (CD8^+^/ CD90.1^+^) proliferation and specific cytotoxic killing of target cells were measured overnight by spleen analysis. Data are from a single experiment representative of three experiments with each dot representing one mouse. **p < 0.01, ***p <0.001.

Beside the prolonged antigen presentation in vivo, we determined whether these antigen containing resident APCs were able to induce functional CD8^+^ T cells with killing capacity. C57BL/6 mice that have been injected with OVA specific antibody followed by OVA antigen received OTI T‐cell adoptive transfer after 48 h or 1 week (Fig. 2A). The induction of effector CD8^+^ T cells was determined by the decrease of CD127^+^ (IL‐7Rα) and the increase of CD44^+^ expression on OTI T cells (data not shown). To determine whether these T cells were able to kill specific target cells in vivo, splenic target cells were loaded with OVA peptide or control peptide and injected in mice 4 days after OTI adoptive transfer. The next day, in vivo killing of target cells was analyzed in the spleen. Mice that received anti‐OVA antibody and OVA, 48 h prior to the OTI adoptive transfer, showed near 80% specific killing and a higher percentage of OTI cells of total CD8^+^ T cells compared to mice that only received OVA (Fig. 2B). Mice that were injected with OTI cells 1 week after they first received anti‐OVA antibody and OVA also showed higher numbers of OTI cells with almost 60% specific killing of the target cells and no detectable killing in mice that only received soluble OVA (Fig. 2C). Similar results were found when in vivo killing of target cells was analyzed in the lymph nodes derived from the same mice (Supporting Information Fig. 1).

### APC subsets take up and store antigen for several days in vivo

**Figure 3:**
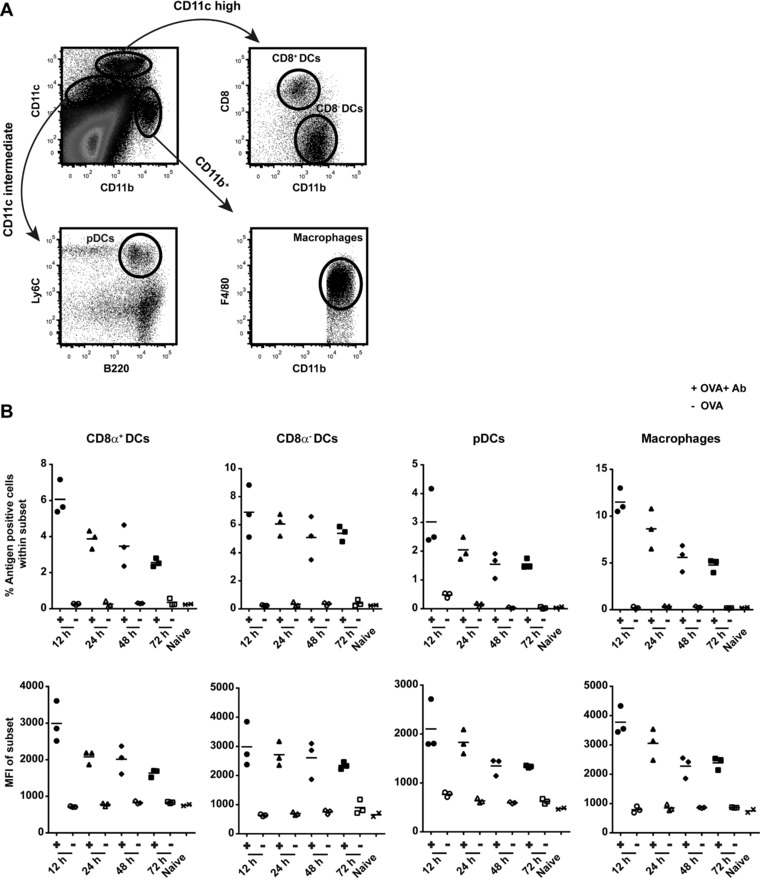
Sustained antigen presence in APC subsets in vivo. Four splenic APC subsets in BL/6 mice were distinguished by the following markers in flow cytometry: CD8α^+^ DCs (CD11c^high^ CD8α^+^ CD11b^−^), CD8α^−^ DCs (CD11c^high^ CD8α^−^ CD11b^+^), pDCs (CD11c^int^ Ly6C^+^ B220^+^) and macrophages (CD11c^int^ CD11b^+^ F4/80^+^) (A). BL/6 mice were injected with Ab i.v. followed by OVA (Alexa Fluor 647 labeled) i.v. injection 30 min later. Antigen presence in spleens (each dot represents one mouse) was analyzed after 12 h/24 h/48 h/72 h, indicated by percentage positive cells and mean fluorescence intensity (MFI) of Alexa Fluor 647 (B). Flow cytometry data are from a single experiment with three mice per group, representative of four independent experiments.

We have previously shown that CD11c^+^ cells are crucially involved in antigen presentation of in vivo complexed antigen 12. Since we observed prolonged T‐cell activation, proliferation and killing capacity above, we set out to determine which APC subsets play a role in sustained antigen storage in vivo. Four subsets in the spleen of mice were distinguished by the following markers: CD8α^+^ DCs (CD11c^high^ CD8α^+^ CD11b^−^), CD8α^−^ DCs (CD11c^high^ CD8α^−^ CD11b^+^), pDCs (CD11c^int^ Ly6C^+^ B220^+^) and macrophages (CD11c^int^ CD11b^+^ F4/80^+^) using flow cytometry (Fig. 3A). The major population is the macrophages (∼3% of total spleen cells), followed by CD8α^−^ DCs (∼2%), CD8α^+^ DCs (∼1%) and pDCs (<0.5%). Mice were injected sequentially with anti‐OVA IgG antibody and Alexa Fluor 647 labeled OVA to track the uptake in the APC subsets. Approximately 6% of the CD8α^+^ DCs were antigen positive after 12 h of antibody and OVA injection (Fig. 3B upper panel, Supporting Information Fig. 2A). This remains sustained in time up to 72 h where 2% antigen positive CD8α^+^ DCs were found in the spleen (Fig. 3B upper panel). A similar pattern of sustained antigen presence in time was found in the other APC subsets. Measuring the mean Alexa 647 fluorescence intensity (MFI) of the subsets showed less than 50% decrease from 12 h up to 72 h after antibody and OVA injection (Fig. 3B lower panel). Mice that only received OVA without antibody did not show any detectable uptake indicating the efficiency of antibody‐mediated uptake of OVA (Fig. 3B, Supporting Information Fig. 2A). Similar uptake with soluble OVA was only reached when 40 times more OVA was given compared to antibody‐mediated uptake (data now shown).

Using an irrelevant antibody against HPV E6 protein we observed no detectable antigen uptake by all APC subsets, indicating that antigen uptake is only achieved with the use of antigen specific antibodies (Supporting Information Fig. 2B). To show that this highly efficient antibody‐mediated antigen uptake was not due to the use of rabbit specific IgG, mice were prime‐boost vaccinated with OVA protein to generate endogenous murine anti‐OVA IgG antibodies (Supporting Information Fig. 2C). Two weeks after the booster vaccination, seropositive mice were injected with OVA (Alexa Fluor 647 labeled) and efficient antigen uptake in all APC subsets was detected (Supporting Information Fig. 2D). Moreover, when serum from OVA‐vaccinated mice, containing anti‐OVA antibodies, was transferred to naïve mice followed by Alexa Fluor 647 labeled OVA injection, antigen uptake was detected in all APC subsets in contrast to control mice (Supporting Information Fig. 2E).

Next we analyzed the presence of OVA protein in serum during our experiments. A possible explanation for the sustained antigen presence in APCs is that OVA protein is remaining in the circulation of the mice which may allow continuous uptake of fresh OVA by APCs over time. This is however an unlikely option since most of the single injection of OVA was cleared from the circulation already after 2 h and was non‐detectable after 24 h (Supporting Information Fig. 3). Taken together, these data demonstrate that APC subsets in vivo have the ability to efficiently engulf and store antigen in an antibody‐dependent fashion for several days.

### Antigen is stored in punctuated compartments in APC subsets in vivo

**Figure 4:**
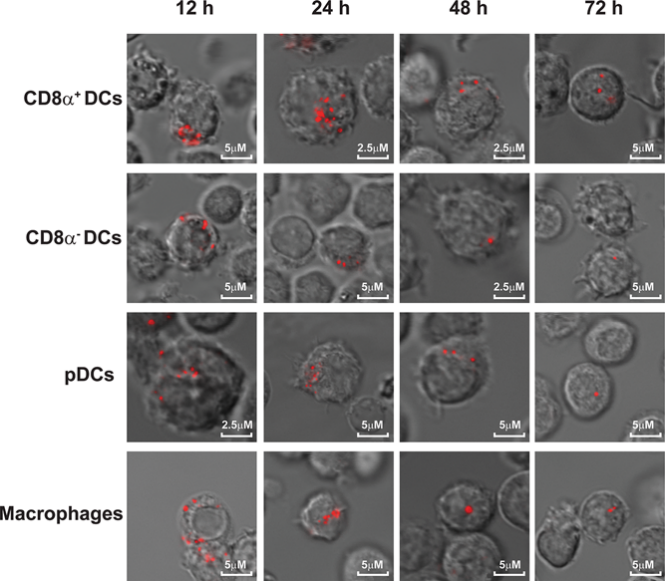
Localization of antigen storage in APC subsets in vivo. BL/6 mice were injected with Ab i.v. followed by OVA (Alexa Fluor 647 labeled) injection after 30 min. Four APC subsets were sorted at different time points according to the markers described in Fig. 3A and antigen presence in live cells was visualized by confocal microscopy. Data are from a single experiment representative of three experiments with two mice per experiment.

In order to visualize and localize where antigen was stored in APC subsets, all four subsets were sorted from the spleen at different time points after the injection of anti‐OVA antibody and Alexa Fluor 647 labeled OVA and analyzed by confocal microscopy. An overlay of the fluorescence signal and differential interference contrast (DIC) observed in optical slices from the ex vivo isolated APC subsets show the location of antigen within the cells. After 12 h of antibody and OVA injection several intensely fluorescent punctuated “hotspots” were found in all APC subsets (Fig. 4). Co‐staining with Lysotracker, a marker for endo‐lysosomal compartments, showed partial co‐localization with OVA indicating intracellular localization and storage of antigen in DCs in endo‐lysosomal compartments (Supporting Information Fig. 4), similar as we described previously for DCs in vitro 1. In general the number of hotspots decreased in time in all four subsets but remained detectable even after 72 h. These results are in line with the MFI detection in Fig. 3B, indicating localized storage and gradual decrease of antigen in time in these APC subsets in vivo.

### Sustained and selective MHC I and II antigen presentation by CD8α^+^ and CD8^−^ DCs

**Figure 5:**
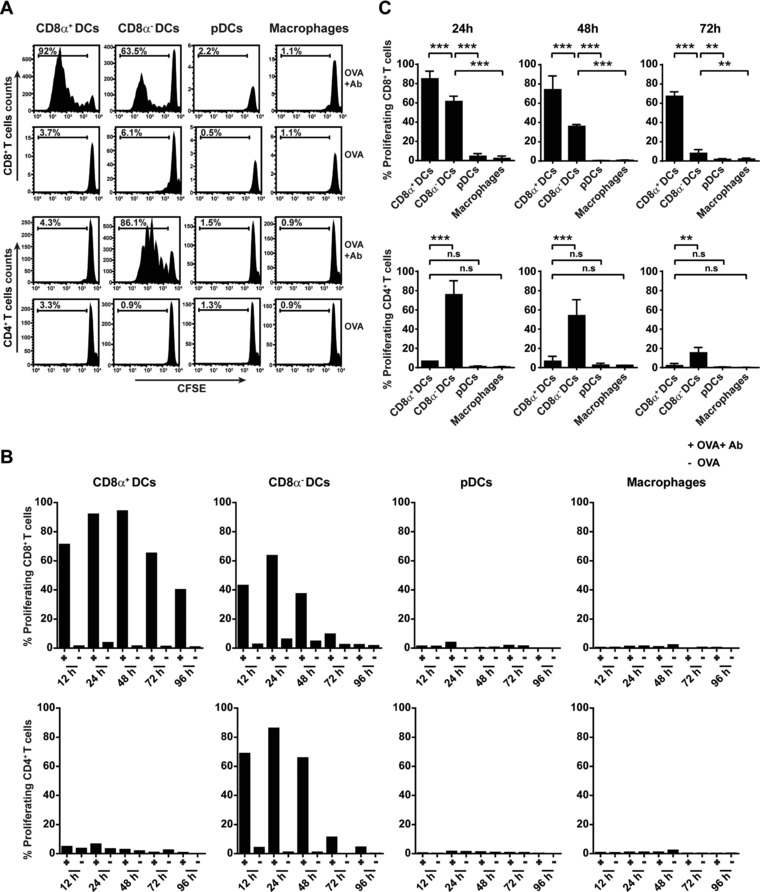
Sustained and selective antigen presentation by splenic CD8α^+^ and CD8α^−^ DCs. BL/6 mice (two mice per group) were injected with Ab followed by OVA i.v. injection 30 min later. At different time points (12 h/24 h/48 h/72 h and 96 h), splenic APC subsets were isolated of individual mice by FACS sorting according to the markers described in Fig. 3A and incubated with CFSE‐labeled OTI (CD8^+^/ CD90.1^+^) or OTII (CD4^+^/ CD45.1^+^) cells ex vivo for 3 days. CD8^+^ T‐cell and CD4^+^ T‐cell proliferation by APC subsets was measured in mice that received OVA and Ab or only OVA for 24 h, indicated by the percentage of divided T cells (A). Percentage of CD8^+^ T‐cell and CD4^+^ T‐cell proliferation activated by APC subsets at all time points is shown from one representative experiment out of three performed (B). Average proliferation data of three independent experiments showing MHCI cross‐presentation (upper panel) and MHCII antigen presentation (lower panel) by each APC subset after 24, 48 and 72 h (C). **p < 0.01, ***p <0.001, n.s: non‐significant.

To address the question which APC subsets contributes to sustained antigen presentation to CD8^+^ and CD4^+^ T cells, mice were injected sequentially with anti‐OVA antibody and OVA. At different time points four APC subsets were separately sorted from the spleen, as described before, and incubated ex vivo with either CD8^+^ OTI or CD4^+^ OTII cells. Both CD8α^+^ DCs and CD8α^−^ DCs cross‐presented antigen to OTI cells, with CD8α^+^ DCs being the most potent cross‐presenting subset (Fig. 5A). On the other hand, CD8α^−^ DCs were the only subset that could present antigen to OTII cells. All four subsets that were isolated from spleens of mice that were injected with the same dose of soluble OVA protein did not show any detectable antigen presentation to either OTI or OTII cells. Following the CD8α^+^ DC subset in time showed their sustained capacity to cross‐present antigen to OTI cells: 40% OTI cells proliferated upon encountering CD8α^+^ DCs that had stored antigen for 96 h (Fig. 5B). Although the CD8α^−^ DCs were able to cross‐present antigen, it was in a lesser extent compared to the CD8α^+^ DCs, e.g. only 10% OTI cells proliferated after being exposed to CD8α^−^ DCs that had stored antigen for 72 h. However, the CD8α^−^ DCs showed potent and sustained antigen presentation to OTII T cells up to 72 h, whereas the CD8α^+^ DCs failed to do so at any time point. In contrast, the pDCs and macrophages could not present antigen to either OTI or OTII cells in any extent. Figure 5C shows significant higher antigen cross‐presentation ability of CD8α^+^ DCs compared to other APC subsets which was most pronounced after 72 h. The CD8α^−^ DCs was the only subset showing significant and sustained antigen presentation to CD4^+^ T cells (Fig. 5C). In order to investigate whether the differences found in antigen presentation ability of the four subsets were due to the lack of MHCI or MHCII expression on their cell surface, spleens from naïve mice were sorted and loaded with antigenic peptides (Supporting Information Fig. 5). All four subsets that were loaded with the MHCI binding peptide OVA8 were fully competent to activate OTI cells. The APC subsets that were loaded with the MHCII binding T helper peptide OVA17 were also able to present in MHCII to OTII cells, although the pDCs and macrophages in a lower degree. Taken together, these data show that all four APC subsets take up and store antigen for several days, but there is a clear functional distinction in their antigen presentation ability.

## Discussion

DCs are a heterogeneous cell population in vivo where each subset has different capacities in antigen‐presentation. We have previously reported that DCs can conserve exogenous protein antigen in a storage organelle, distinct from MHC class II compartments or MHCI processing/ loading compartments, for several days that corresponds with sustained CTL cross‐priming in vivo 1. In the current study we further investigate the antigen storage and presentation abilities of APC subsets (CD8α^+^ DCs, CD8α^−^ DCs, pDCs and macrophages) in vivo. In contrast to most studies that used in vitro preformed Ag‐Ab immune complexes (IC), we sequentially injected mice with antibody followed by cognate protein antigen (i.v.) to allow natural formation of IC in circulation. We show here for the first time that all APC subsets take up antigen and have the ability to store antigen for several days in vivo. This corresponds with long‐lasting in vivo antigen presentation to CD8^+^ and CD4^+^ T cells and effective cytotoxic T‐cell killing of target cells up to a week after antigen injection. From all APC subsets, CD8α^+^ DCs were superior in antigen cross‐presentation and showed sustained CD8^+^ T‐cell activation after isolation. On the other hand, only CD8α^−^ DCs were able to present antigen to CD4^+^ T cells several days after antigen injection. These results are in line with other studies that show distinct antigen presentation capacities by CD8α^+^ DCs and CD8α^−^ DCs to T cells 6, 7, 9, 10, 11. It had been suggested that cross‐presentation by CD8α^−^ DCs depends on activating Fcγ receptors (FcγR), since MHCI antigen presentation by CD8α^−^ DCs was hampered in γ‐chain‐deficient mice 8. Cross‐presentation by CD8α^+^ DCs from γ‐chain‐deficient mice remained intact, indicating that activation of CD8α^+^ DCs is not necessary for efficient cross‐presentation. However, we have recently shown that complement factor C1q, and not FcγR, plays a major role in antibody‐mediated antigen uptake from blood circulation and presentation by APCs in vivo 13. Mice lacking C1q showed no detectable antigen uptake in APCs and strongly reduced antigen presentation to CD8^+^ or CD4^+^ T cells.

Despite that pDCs and macrophages take up antigen, we showed no detectable antigen presentation to T cells. Both pDCs and macrophages have been described as poor antigen cross‐presenting APCs compared to cDCs 14, 15, 16, 17, 18, 19, 20. Several studies have addressed the contribution of pDCs in cross‐priming of CD8^+^ T cells with different infection models since pDCs are unique in their ability to produce large amounts of type I interferons (IFN I) upon viral infections, however no specific role for pDCs was found in MHCI cross‐presentation 21, 22. pDCs probably play a more important role in endogenous viral infections or there still might be unidentified specific antigen targeting routes that can induce the cross‐presentation ability of pDCs. Macrophages mostly function as the first line of defense against pathogens by its rapid particle and immune complex removal and degradation abilities. One study showed that macrophages were able to prime naïve T cells in vivo by using 10‐fold more antigen compared with DCs 23. Based on our data we however feel that macrophages are far less effective in T‐cell priming than DCs. Although it has been suggested that macrophages have more acidic endosomal environments with many lysosomal proteases that results in faster antigen degradation compared to DCs 24, 25, we could still detect similar amounts of stored protein antigen in splenic macrophages compared to other DC subsets 3 days after antigen and antibody injection. Moreover, others have shown that the antigen uptake capacity does not determine cross‐presentation efficiency in APC subsets 11. Therefore it is more likely that, at least in this targeting system, other mechanisms located downstream from antigen uptake and storage determine the cross‐presentation outcome of APC subsets.

We have previously demonstrated that the processing of antigen from the storage organelle in DCs involves a cytosolic and TAP‐dependent pathway since inhibiting proteasomal activity or using TAP deficient DCs almost completely inhibits MHCI cross‐presentation 1. Therefore, it is presumable that antigens from the storage compartment are degraded by the proteasome in the cell cytosol and loaded on MHCI in the ER, although it is not excluded that peptides, after proteasome degradation, are transported back into endocytic compartments where they are trimmed by IRAP and loaded on MHCI as reported by Van Endert and colleagues 26. It is still unclear how antigens are transferred from endosomes into the cytosol for proteasomal degradation. One study used exogenous cytochrome c, which induces apoptosis upon release in the cell cytosol, to show that CD8^+^ DCs was the major DC subset reduced by the treatment in vivo 27. These data suggest that CD8^+^ DCs possess a highly efficient mechanism for endosome‐to‐cytosol transport compared to other APC subsets. Some reports suggested Sec61 as the translocator for endosome‐to‐cytosol transport of antigens for DC cross‐presentation to CD8^+^ T cells 28, 29, however a more recent study showed the opposite 30. Others showed that by silencing Sec22b, an ER‐resident SNARE, cross‐presentation by DCs is impaired due to antigen transfer inhibition from phagosomes to the cytosol 31. Nevertheless, it is still not clear which specific translocator on DC endosomal membranes regulate antigen transportation from endosomes to the cytosol. Characterizing translocator function might give better insight into the different cross‐presentation outcome of APC subsets.

We have shown previously that cell surface MHCI on DCs have a shorter turnover rate compared to MHCII 1. Most MHCI‐peptide complexes disappear from the cell surface within 24 h. Since the migration of DCs after antigen encountering to the T‐cell zones might take up to several days, this high turnover rate of MHCI is not beneficial for efficient CD8^+^ T‐cell cross‐presentation 32. Also the dose of antigen that is expressed on MHCI needs to exceed the required threshold for effective T‐cell activation. Therefore, long term antigen storage in DCs and sustained antigen display on the DC cell‐surface is important to ensure T‐cell cross‐priming. Several reports have shown that DCs in vivo have relative rapid turnover rates 33, 34, 35. It has been suggested that cDCs in mouse spleen undergo faster turnover and have a shorter lifespan than pDCs, approximately a half‐life of 2 days and 8 days respectively. Some studies suggest that rapid turnover of cDCs favors the engulfment by other DCs which facilitate antigen processing and presentation 36, 37. In regard to our observations of sustained in vivo antigen presentation of splenic APCs, the significance of different lifespans of APC subsets may also contribute to modulation of immune control.

In conclusion, we show here for the first time that in vivo DC subsets can store antigen for several days in storage compartments and that this contributes to sustained and selective antigen cross‐presentation to CD8^+^ and CD4^+^ T cells. Further characterization of the downstream antigen processing pathways within each APC subset may reveal why specific subsets are superior in MHCI cross‐presentation or MHCII antigen presentation.

## Materials and methods

### Mice

All animal experiments in this paper have been approved by the review board of Leiden University Medical Center. C57BL/6 mice were purchased from Charles River Laboratories. CD45.1 congenic mice on C57BL/6 background, OTI mice (CD8^+^ T‐cell transgenic mice expressing a TCR recognizing the OVA derived K^b^ associated epitope SIINFEKL) and OTII mice (CD4^+^ T‐cell transgenic mice expressing a TCR recognizing the OVA derived Th epitope ISQAVHAAHAEINEAGR in association with I A^b^) were bred and kept at the LUMC animal facility under SPF conditions. All mice were used at 8–12 weeks of age.

### In vivo formed OVA‐IgG complexes

C57BL/6 mice were intravenously injected with 100 μg polyclonal rabbit anti‐OVA IgG (ICN Biomedicals). After 30 min of antibody circulation, mice were injected intravenously with 5 μg Ovalbumin (OVA, Worthington Biochemical Corporation) or OVA conjugated with Alexa Fluor 647 (Life Technologies). As control antibody, polyclonal rabbit anti‐human papilloma virus (HPV) E6 IgG were generated in our lab by vaccinating New Zealand rabbits with recombinant E6 protein.

### T‐cell proliferation in vivo

Recipient mice received intravenously CFSE‐labeled spleen cells from OTI or OTII mice (purified with CD8 and CD4 T lymphocyte enrichment kit respectively, BD Biosciences). After 4 days, spleens from the recipient mice were collected and proliferation of CFSE‐ labeled T lymphocytes was analyzed by flow cytometry. OTI cells were gated as CD8^+^/ CD90.1^+^ cells. OTII cells were gated as CD4^+^/ CD45.1^+^ cells.

### In vivo cytotoxicity assay

C57BL/6 mice were injected with anti‐OVA antibody and OVA to form OVA‐IgG complexes in vivo as described before or only with OVA. After different time points mice received spleen cells from OTI mice (CD8^+^ T‐cell enriched) intravenously. 4 days later Ly5.1^+^ splenocytes were used at target cells. The target cells were labeled with 5 μmol/L CFSE and pulsed with OVA peptide SIINFEKL (0.5 μg/mL, 1 h at 37°C), or labeled with 0.5 μmol/L CFSE and pulsed with the control D^b^‐ binding flu‐peptide ASNENMDAM (1 μg/mL, 1 h at 37°C). The target cells were mixed 1:1 ratio and injected intravenously. Spleen cells and lymph nodes were isolated the next day and the number of specific CFSE^high^ and control CFSE^lo^ Ly5.1^+^ were measured by flow cytometry. CD8^+^/ CD90.1^+^ OTI cells were stained for effector T‐cell markers CD127 and CD44. The percentage of specific killing is calculated as follows: (1‐(ratio injected mice/ ratio background mice)) × 100%. Ratio is defined by the number of SIINFEKL CFSE^high^ target cells/ number of control CFSE^low^ target cells. Background mice are C57BL/6 mice injected with only OTI cells.

### Ex vivo antigen presentation

Spleens from C57BL/6 mice with in vivo formed OVA‐IgG complexes or only OVA were isolated at different time points. APC subsets sorting was performed on BD FACSAria II SORP (BD Biosciences). The purity of each subset after the sort was determined by flow cytometry: CD8α^+^ DCs (86%), CD8α^−^ DCs (81%), pDCs (50%) and macrophages (67%). Contaminations by other subsets were less than 1%. Each APC subset (50.000 cells) was incubated with CFSE labeled and purified OTI (50.000) or OTII cells (50.000) in a 96 well round bottom plate. CD8^+^ and CD4^+^ T‐cell proliferation was measured after 4 days by flow cytometry. Minimal peptide loading controls were performed with APC subsets from naïve C57BL/6 mice. After isolation, each subset was incubated with 100 pg/mL OVA8 (SIINFEKL) or 1 μg/mL OVA17 (ISQAVHAAHAEINEAGR) for 1 h and washed extensively afterwards. Each subset was then incubated with either CFSE labeled OTI or OTII cells and T‐cell proliferation was measured by flow cytometry 3 days later.

### Antigen presence in splenic APC subsets

C57BL/6 mice with in vivo formed OVA‐IgG complexes or only OVA (Alexa Fluor 647 labeled, Life Technologies) were sacrificed at different time points. Spleens were isolated and dissociated with Liberase (Thermolysin Low, research grade, Roche) for 20 min at 37°C. The antigen presence was measured by the percentage of Alexa Fluor 647 positive cells and the mean fluorescence intensity (MFI) within each APC subset. Background levels were determined by naïve mice without any injections.

In addition, antigen presence in splenic APC subsets was visualized by confocal scanning laser microscopy. APC subsets sorting was performed as described before and sorted cells were incubated for 30 min with Lysotracker (Green DND‐26, ThermoFisher) at 37°C, washed and transferred to glass bottom dishes (MatTek coporation, Ashland, USA). Live cells were imaged using Leica SP5 STED confocal microscope with a 63x objective lens. Differential interference contrast (DIC) was additionally used to image cell contrast. Images were acquired by taking optical slices in 10× magnification and were processed with Leica LAS AF software. Co‐localization was measured by a line scan drawn on a single optical slice and plotted in histograms. Each arrow indicates co‐localization between OVA and Lysotracker.

### Ovalbumin specific mouse IgG generation and transfer

C57BL/6 mice were injected s.c. with 100 μg OVA emulsified in Incomplete Freund's Adjuvant (IFA) and boosted 2 weeks later with 100 μg OVA in IFA. After two weeks, blood was withdrawn from the lateral tail vein and the presence of mouse anti‐OVA IgG was determined by ELISA. Seropositive C57BL/6 mice were injected i.v. with 5 μg OVA (Alexa Fluor 647 labeled) and spleen APCs were analyzed 24 h later for the presence of fluorescent OVA by flow cytometry. Furthermore, sera from OVA seropositive C57BL/6 mice were collected and transferred i.v. into naïve C57BL/6 mice followed by i.v. injection of 5 μg OVA (Alexa Fluor 647 labeled). The presence of fluorescent OVA was measured 24 h later in spleen APCs by flow cytometry.

### Quantification of ovalbumin in mouse serum

Naïve C57BL/6 mice were injected i.v. with 100 μg polyclonal rabbit anti‐OVA IgG. After 30 min, mice were injected i.v. with 5 μg OVA (Alexa Fluor 647 labeled). At indicated time points 50 μL blood was withdrawn from the lateral tail vein and serum was collected. A total of 5 μL serum was mixed with sample buffer, heated at 95°C for 5 min and loaded on SDS/PAGE. Fluorescent OVA was quantified directly from the SDS/PAGE gels by using a Typhoon 9410 Variable mode imager (GE Healthcare Bio‐Sciences) and ImageQuant TL v8.1 software (GE Healthcare Life Sciences), indicated by relative pixel intensity.

### Statistical analysis

Statistical analysis was performed using one‐way analysis of variance (ANOVA) test. Tukey's *post hoc* test was performed to correct for multiple comparisons The following indications are used in all figures: *p <0.05, **p < 0.01, ***p <0.001, n.s: non‐significant.

## Conflict of interest

The authors have no financial or commercial conflicts of interest.

**Abbreviations**
APCs: antigen presenting cells
cDCs: conventional DCs
DCs: dendritic cells
MFI: mean fluorescence intensity
OVA IC: OVA immune complexes
OVA: ovalbumin
pDCs: plasmacytoid DCs

## Supporting information

Supporting Information

Supporting Information
