# Supplementary material for: Sustained cross‐presentation capacity of murine splenic dendritic cell subsets in vivo
Source: Eur J Immunol. 2018 May 17;48(7):1164–73. doi: 10.1002/eji.201747372 (PMC6055716; doi:10.1002/eji.201747372)
Supplement: Supplementary file 1 — Supporting Information [file EJI-48-1164-s001.pdf]

European Journal of Immunology

Supporting Information
for
DOI 10.1002/eji.201747372

Nataschja I. Ho, Marcel G. M. Camps, Edwin F. E. de Haas and Ferry Ossendorp

Sustained cross-presentation capacity of murine splenic dendritic cell subsets in vivo

© 2018 The Authors. *European Journal of Immunology* published by WILEY-VCH Verlag GmbH & Co. KGaA, Weinheim.

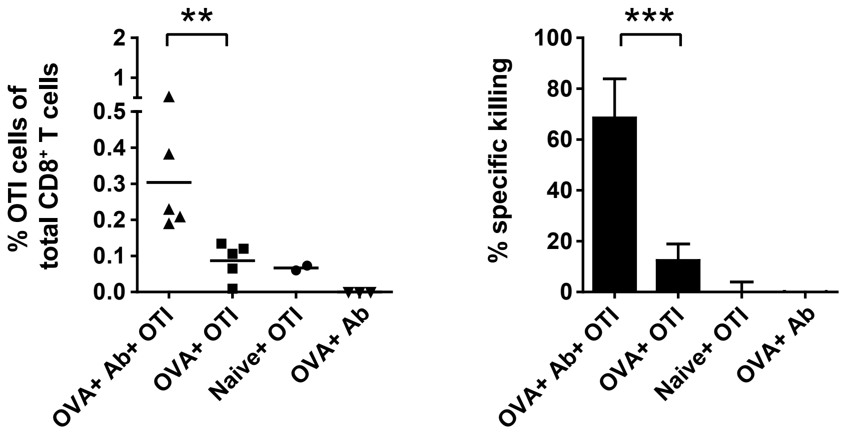

Supplemental Figure 1. Sustained induction of cytotoxic T cell activity in lymph nodes *in vivo*. BL/6 mice were injected i.v. with Ab followed 30 min later by OVA i.v. injection. After 1 week, mice received i.v. adoptive transfer of OTI cells, followed by target cells 4 days later. OTI cell proliferation and specific cytotoxic killing of target cells were measured overnight in the inguinal lymph nodes. Data are from a single experiment representative of three experiments with each dot representing one mouse. $^{**}p < 0.01$, $^{***}p < 0.001$.

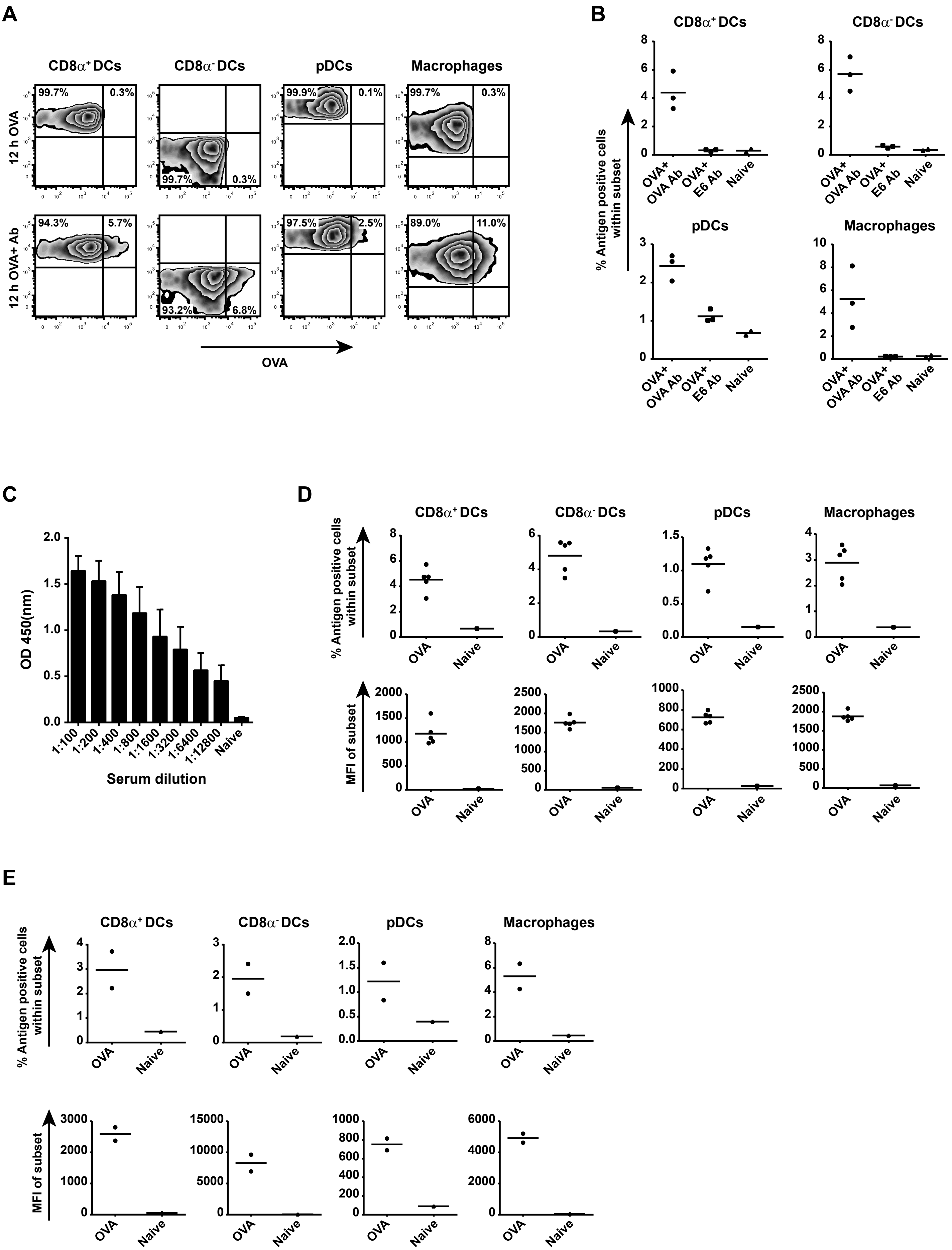
A
CD8α⁺ DCs
CD8α⁻ DCs
pDCs
Macrophages
12 h OVA
12 h OVA+ Ab
99.7%
0.3%
99.9%
0.1%
94.3%
5.7%
93.2%
6.8%
97.5%
2.5%
89.0%
11.0%
OVA
B
% Antigen positive cells within subset
OVA+ OVA Ab
OVA+ E6 Ab
Naive
C
OD 450(nm)
1:100
1:200
1:400
1:800
1:1600
1:3200
1:6400
1:12800
Naive
Serum dilution
D
OVA
MFI of subset
E

Supplemental Figure 2. Endogenous mouse anti-OVA IgG mediates efficient OVA uptake in splenic APC subsets. BL/6 mice were injected and analyzed as described in Fig.3 (A). BL/6 mice (each dot represents one mouse) were injected with 100 µg polyclonal rabbit anti-OVA IgG or with 100 µg anti-HPV E6 antibody followed 30 minutes later by 5 µg OVA (Alexa Fluor 647 labeled). After 24 h the presence of OVA was measured in splenic APC subsets by flow cytometry (B).

BL/6 mice (N=5) were vaccinated with OVA s.c. and boosted 2 weeks later with OVA. After two weeks, blood was taken from mice and the presence of anti-OVA in mouse serum was determined by ELISA (C). Seropositive BL/6 mice (each dot represents one mouse) were i.v. injected with OVA (Alexa Fluor 647 labeled) and the presence of OVA was measured in splenic APC subsets after 24 h by flow cytometry (D). Serum containing anti-OVA IgG from OVA-vaccinated BL/6 mice was transferred i.v. into naïve BL/6 mice followed by i.v. injection of OVA (Alexa Fluor 647 labeled). The presence of OVA was measured 24 h later by flow cytometry (E).

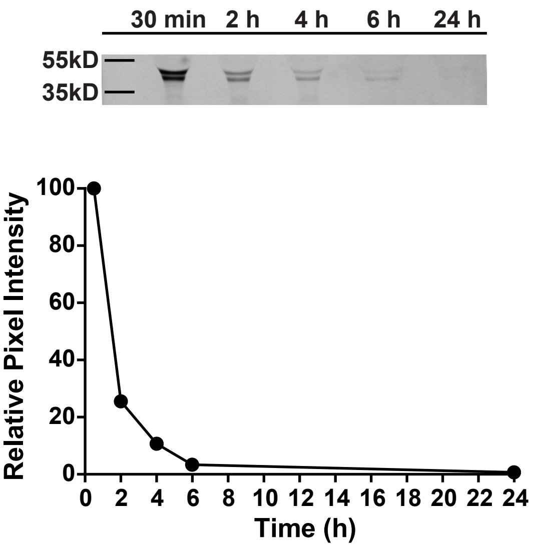

Supplemental Figure 3. Rapid clearance of OVA in Ab positive mice. BL/6 mice received polyclonal rabbit anti-OVA IgG i.v. followed 30 minutes later by OVA (Alexa Fluor 647 labeled). At different time points serum was collected and the presence of fluorescent OVA was quantified from SDS/PAGE gels and indicated by relative pixel intensity. Data are from a single experiment representative of two experiments.

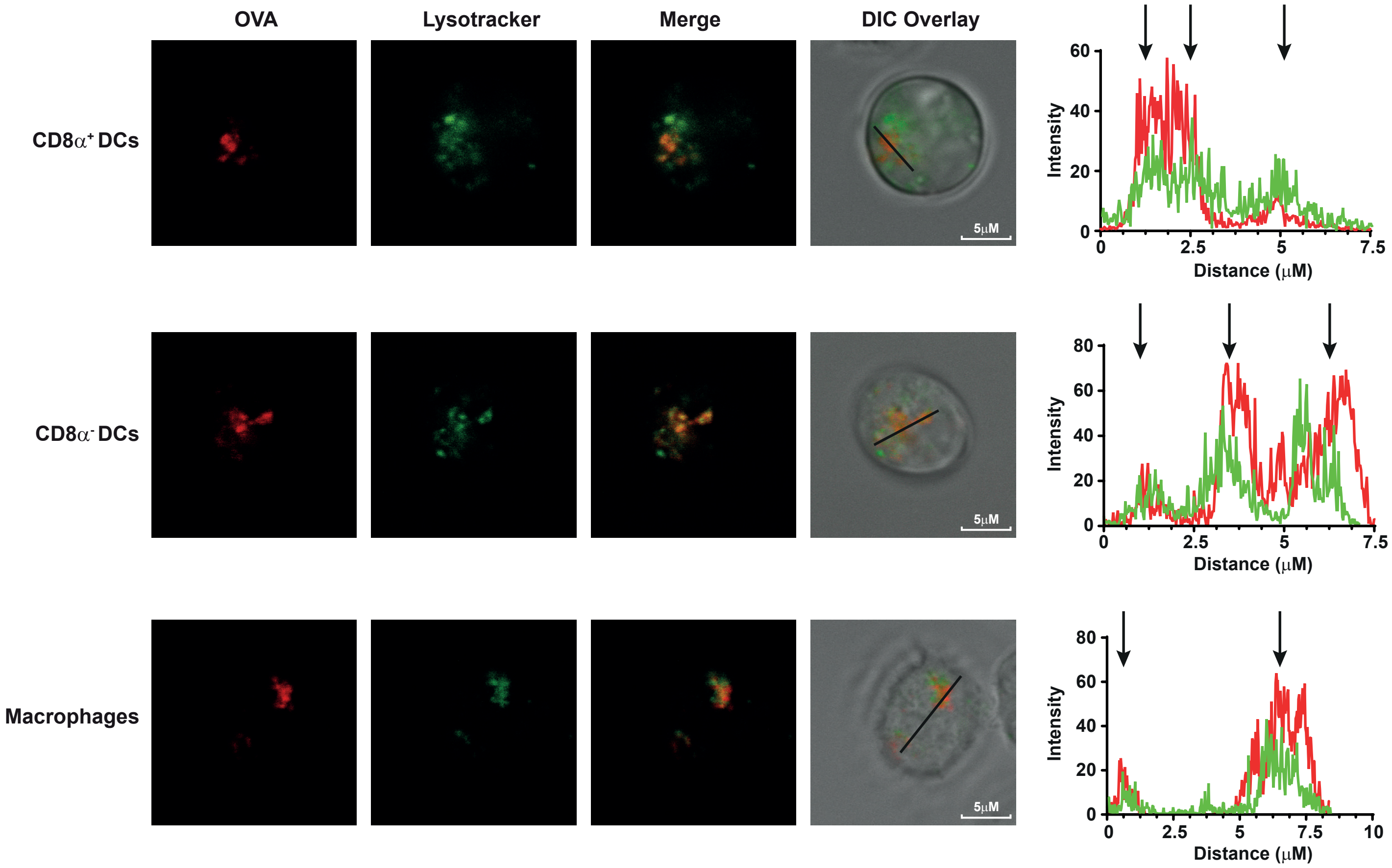

Supplemental Figure 4. Intracellular localization of antigen storage. Experiment was performed as described in Fig. 4. After sorting the splenic APC subsets 24 h after injection of Ab and OVA (Alexa Fluor 647 labeled) , Lysotracker Green was added for 30 min *ex vivo* and co-localization with OVA antigen in live cells was visualized by confocal microscopy. Co-localization was analyzed by a line scan drawn on a single optical slice and plotted in histograms. Arrows indicate co-localization between OVA and Lysotracker.

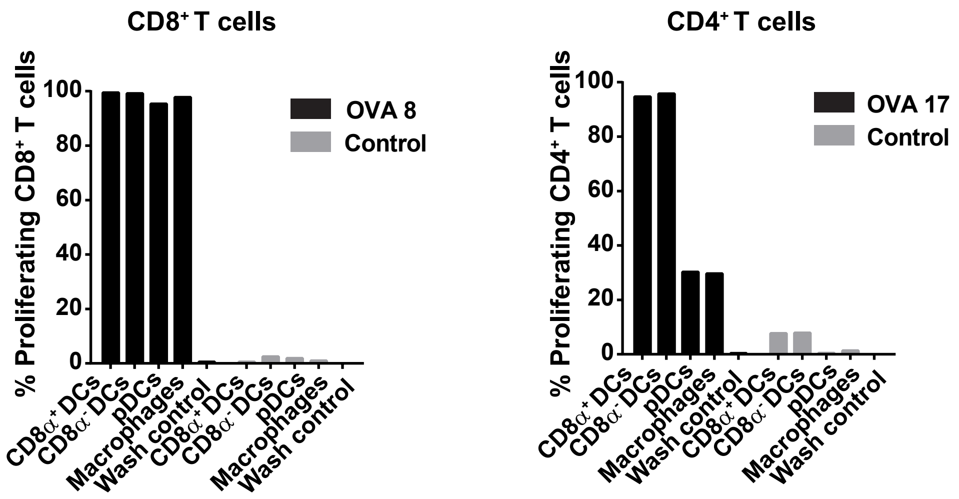

Supplemental Figure 5. All four APC subsets are competent in MHCI and MHCII antigen presentation. Four splenic APC subsets from naïve BL/6 mice were sorted according to the markers described in Fig. 3A and incubated with OVA8 (SIINFEKL) or OVA17 (ISQAVHAAHAEINEAGR) synthetic peptides. After extensively washing, each APC subset was incubated with CFSE-labeled OTI ($CD8^+$/ $CD90.1^+$) or OTII ($CD4^+$/ $CD45.1^+$) cells. T cell proliferation was measured after 3 days by flow cytometry. Wash controls were done without the presence of cells. Data are from a single experiment representative of two experiments.
