# Supplementary material for: Sustained cross‐presentation capacity of murine splenic dendritic cell subsets in vivo
Source: Eur J Immunol. 2018 May 17;48(7):1164–73. doi: 10.1002/eji.201747372 (PMC6055716; doi:10.1002/eji.201747372)
Supplement: Supplementary file 2 — Supporting Information [file EJI-48-1164-s002.pdf]

European Journal of Immunology

Supporting Information

for

DOI 10.1002/eji.201747372

Nataschja I. Ho, Marcel G. M. Camps, Edwin F. E. de Haas and Ferry Ossendorp

Sustained cross-presentation capacity of murine splenic dendritic cell subsets in vivo

© 2018 The Authors. *European Journal of Immunology* published by WILEY-VCH Verlag GmbH & Co. KGaA, Weinheim.

Sustained cross-presentation capacity of murine splenic dendritic cell subsets in vivo

Nataschja I. Ho, Marcel G. M. Camps, Edwin F. E. de Haas and Ferry Ossendorp

Correspondence: Dr. Ferry Ossendorp

Department of Immunohematology and Blood Transfusion, Leiden University Medical Center, Leiden, The Netherlands

---

Review Timeline:	Submission date:	13-Oct-2017
	First Editorial decision:	06-Nov-2018
	Revision received:	24-Jan-2018
	Second Editorial decision:	01-Feb-2018
	Second Revision received:	22-Mar-2018
	Accepted:	17-Apr-2018

Handling Executive Committee member: Prof. Kenneth Murphy

Please note that the correspondence below does not include the standard editorial instructions regarding preparation and submission of revised manuscripts, only the scientific revisions requested and addressed.

First Editorial Decision

06-Nov-2018

06-Nov-2017

Dear Dr. Ossendorp,

Manuscript ID eji.201747372 entitled "Sustained cross-presentation capacity of splenic dendritic cell subsets in vivo" which you submitted to the European Journal of Immunology has been reviewed. The comments of the referees are included at the bottom of this letter.

A revised version of your manuscript that takes into account the comments of the referees will be reconsidered for publication. The Executive Editor encourages you to onboard the comments of the referees and should you disagree with any of the referees' concerns, you should address this in your

point-by-point response and provide solid scientific reasons for why you will not make the requested changes.

You should also pay close attention to the editorial comments included below. **In particular, please edit your figure legends to follow Journal standards as outlined in the editorial comments and state the number of independent experiments performed in the figure legends. Failure to do this will result in delays in the re-review process.**

Please note that submitting a revision of your manuscript does not guarantee eventual acceptance, and that your revision will be re-reviewed by the referee(s) before a decision is rendered.

If the revision of the paper is expected to take more than three months, please inform the editorial office. Revisions taking longer than six months may be assessed by new referee(s) to ensure the relevance and timeliness of the data.

Once again, thank you for submitting your manuscript to European Journal of Immunology and we look forward to receiving your revision.

Yours sincerely,
Eloho Etemire

On behalf of
Prof. Kenneth Murphy

Dr. Eloho Etemire
Editorial Office
European Journal of Immunology
e-mail: ejied@wiley.com
www.eji-journal.eu

****

European Journal of Immunology

Reviewer: 1

Comments to the Author

In this manuscript, the authors examine antigen cross-presentation in vivo over time after formation of immune complexes. This study is of interest, however there are a number of concerns that need to be addressed to reinforce the authors' conclusions.

Major concerns

1. In all figures, the authors show "representative results" and it seems that they performed statistical analysis on replicates from the same experiment. In order to evaluate the reproducibility of the results, it is essential that the authors show all data, pooled from the different independent experiments. They should perform statistical analysis on all pooled results.
2. A critical control experiment is missing. The authors claim that they examine cross-presentation of immune complexes that were formed in vivo. However, an other interpretation is that the injection of antibodies could activate DC, and thereby boost their cross-presentation ability. The authors need to compare cross-presentation in mice injected with anti-OVA antibodies versus a similar dose of irrelevant antibodies.
3. The authors claim that immune complexes are stored in APC in vivo. However, the images shown in figure 4 do not show intracellular localisation. An other possibility is that the immune complexes detected by microscopy are present at the cell surface. To reinforce their conclusion, the authors need to provide evidence of the intracellular localisation of the antigen by performing additionnal stainings for intracellular compartments. Even if they have already analysed this phenomenon in cultured DC, it could be different in vivo and they need to provide more evidence or rephrase their conclusions. In addition, it is necessary to provide quantification of the microscopy results.

Other concerns

1. In figure 1, the authors need to indicate how many independent experiments were performed.
2. In figure 2, it is not clear what is depicted as "% OT-I cells of total CD8 T cells". Have the authors performed tetramer staining ?
3. In figure 3, the authors should show a representative FACS histogram of antigen uptake for each population.
4. In figure 4, scale bars are missing.

Reviewer: 2

Comments to the Author

The manuscript by Ho et. al. assesses the longevity of antigen storage and MHC I and MHC II presentation by different dendritic cell populations. This is an interesting manuscript that describes findings of significance to the antigen presentation field. The manuscript is well written, logical and concise. The experiments are well performed and controlled. Addressing the following would enhance the manuscript prior to publication.

The authors should determine the longevity of the CD8 and CD4 T cell division and CTL killing in response to IC-OVA to determine when these responses recede to undetectable levels. While it appears that CD4 T cell division is undetectable after one week, the CD8 T cell responses are still detectable. It would be interesting to know for how long these responses continue in the immunised host.

Figure 3A and B should include (as Supplementary data) representative dot plots to show the signal elicited by OVA-647 for each antigen presenting cell subset.

Figure 4. How can the authors rule out that the antigen is not remaining (in patches) at the surface of the cells examined? Co-localisation of antigen with different markers of intracellular organelles is of interest to delineate the characteristics of the antigen storage compartment.

Figure 5C. Statistical analysis is required. More detail in the legend is required as to what data the graph is displaying (pooled data?).

Could the results obtained in Figure 5C be due to the relative survival of the different cell types in in vitro conditions? In other words is it possible that pDC die rapidly following ex vivo isolation whereas the other DC subsets are more robust? This would lead to the ability of pDC to store and present antigen to be overlooked.

The authors should discuss their findings in the context of what is known about the different antigen presenting cell subsets lifespans and turnover.

First Revision – authors' response

24-Jan-2018

Point-by-point response

Reviewer: 1 Comments to the Author In this manuscript, the authors examine antigen cross-presentation in vivo over time after formation of immune complexes. This study is of interest, however there are a number of concerns that need to be addressed to reinforce the authors' conclusions. Major concerns 1. In all figures, the authors show "representative results" and it seems that they performed statistical analysis on replicates from the same experiment. In order to evaluate the reproducibility of the results, it is essential that the authors show all data, pooled from the different independent experiments. They should perform statistical analysis on all pooled results.

- In all flow cytometry experiments we are showing results that were measured from individual mice, therefore in vivo variation is inevitable. Each experiment has been repeated several times (as indicated in the legends) and showed similar results based on analysis of at least 1x106 ex vivo acquired antigen presenting cells. However, due to inter-assay variations, pooling different independent experiments was not applicable for adequate statistical analysis in this experimental set-up.

2. A critical control experiment is missing. The authors claim that they examine cross-presentation of immune complexes that were formed in vivo. However, an other interpretation is that the injection of antibodies could activate DC, and thereby boost their cross-presentation ability. The authors need to compare cross-presentation in mice injected with anti-OVA antibodies versus a similar dose of irrelevant antibodies.

- We have previously published in this setting that enhanced OVA-specific CD8+ T-cell proliferation was observed in mice that received polyclonal OVA-specific rabbit IgG compared with that of mice that were injected with rabbit control serum, showing that irrelevant Ab does not increase T-cell proliferation (Montfoort et al., Eur. J. Immunol. 2012. 42:598-606.). Now we have also added an additional control experiment figure as Supplemental Figure 2B comparing the uptake of OVA in the presence of circulating rabbit anti-OVA IgG and an irrelevant anti-HPV E6 Ab. Here we show no detectable OVA uptake in all APC subsets when this irrelevant Ab was used.

3. The authors claim that immune complexes are stored in APC in vivo. However, the images shown in figure 4 do not show intracellular localisation. An other possibility is that the immune complexes detected by microscopy are present at the cell surface. To reinforce their conclusion, the authors need to provide evidence of the intracellular localisation of the antigen by performing additionnal stainings for intracellular compartments. Even if they have already analysed this phenomenon in cultured DC, it could be different in vivo and they need to provide more evidence or rephrase their conclusions. In addition, it is necessary to provide quantification of the microscopy results.

- It is technically very difficult to analyze and do co-staining in the ex vivo isolated cells due to the very low harvest numbers of cells after the sorting process. However, the images shown in figure 4 are from optical slices made within one cell with a thickness of 0.5μM. Therefore these images do not show cell surface signals but intracellular fluorescence signals from immune complexes. Quantification of the fluorescence signals (done as reported previously by van Montfoort et al PNAS 2009) show approximately 100 fold higher signal compared to the membrane area, therefore highly suggesting the signal is intracellular. We have adapted the text in the manuscript to better clarify our interpretation.

Other concerns 1. In figure 1, the authors need to indicate how many independent experiments were performed.
- Additional information is added to the figure legends

2. In figure 2, it is not clear what is depicted as "% OT-I cells of total CD8 T cells". Have the authors performed tetramer staining ?
- We injected mice with OT-I cells which can be followed by co-staining CD8+ T cells with the congenic marker CD90.1+, therefore selective gating of the transferred OT-I cell population in recipient CD90.2+ C57BL/6 mice can be achieved without the requirement of tetramer staining.

3. In figure 3, the authors should show a representative FACS histogram of antigen uptake for each population.
- Additional FACS plots are added as requested in Supplemental Fig. 2A.

4. In figure 4, scale bars are missing.
- We have added scale bars to the figure.

Reviewer: 2 Comments to the Author The manuscript by Ho et. al. assesses the longevity of antigen storage and MHC I and MHC II presentation by different dendritic cell populations. This is an interesting manuscript that describes findings of significance to the antigen presentation field. The manuscript is well written, logical and concise. The experiments are well performed and controlled. Addressing the following would enhance the manuscript prior to publication. The authors should determine the longevity of the CD8 and CD4 T cell division and CTL killing in response to IC-OVA to determine when these responses recede to undetectable levels. While it appears that CD4 T cell division is undetectable after one week, the CD8 T cell responses are still detectable. It
would be interesting to know for how long these responses continue in the immunised host. Figure 3A and B should include (as Supplementary data) representative dot plots to show the signal elicited by OVA-647 for each antigen presenting cell subset.
- Additional FACS plots are added as requested in Supplemental Fig 2A.

Figure 4. How can the authors rule out that the antigen is not remaining (in patches) at the surface of the cells examined? Co-localisation of antigen with different markers of intracellular organelles is of interest to delineate the characteristics of the antigen storage compartment.

- It is technically very difficult to analyze and do co-staining in the ex vivo isolated cells due to the very low harvest numbers of cells after the sorting process. However, the images shown in figure 4 are from optical slices made within one cell with a thickness of 0.5μM. Therefore these images do not show cell surface signals but intracellular fluorescence signals from immune complexes. Quantification of the fluorescence signals (done as reported previously by van Montfoort et al PNAS 2009) show approximately 100 fold higher signal compared to the membrane area, therefore highly suggesting the signal is intracellular. We have adapted the text in the manuscript to better clarify our interpretation.

Figure 5C. Statistical analysis is required. More detail in the legend is required as to what data the graph is displaying (pooled data?).

- The data shown in Fig 5C is not pooled. Analysis of every time point is done with antigen presenting cells after extensive sorting of an individual mouse. Due to technical demanding and low harvesting numbers of cells after each sort, it was not possible to have multiple mice for each time point within one experiment to perform statistical analysis. However, the same experiment has been repeated with similar results for each APC subset as indicated in the legend.

Could the results obtained in Figure 5C be due to the relative survival of the different cell types in in vitro conditions? In other words is it possible that pDC die rapidly following ex vivo isolation whereas the other DC subsets are more robust? This would lead to the ability of pDC to store and present antigen to be overlooked.

- To exclude this we have performed a control experiment where we loaded each APC subset with minimal OVA8 peptide which binds directly to surface MHCI (Supplemental Fig. 4). Here we show that the antigen presentation ability of the different cell types are similar in in vitro conditions, indicating that the viability of the cell types are comparable and not affecting the outcome of antigen presentation.

The authors should discuss their findings in the context of what is known about the different antigen presenting cell subsets lifespans and turnover.

- We thank the reviewer for this useful remark and we have added additional remarks on DC subset lifespan in relation to immune control in the discussion section.

European Journal of Immunology

Second Editorial Decision
01-Feb-2018

Dear Dr. Ossendorp,

Thank you for submitting your revised manuscript ID eji.201747372.R1 entitled "Sustained cross-presentation capacity of splenic dendritic cell subsets in vivo" to the European Journal of Immunology. Your manuscript has been re-reviewed and the comments of the referees are included at the bottom of this letter.

Unfortunately, Referee 2 was not satisfied with the revisions made and further major revision is requested. Our Executive Committee has the following requests related to the issues raised by Referee 2.

1. This comment has been ignored:
"The authors should determine the longevity of the CD8 and CD4 T cell division and CTL killing in response to IC-OVA to determine when these responses recede to undetectable levels. While it appears that CD4 T cell division is undetectable after one week, the CD8 T cell responses are still detectable. It would be interesting to know for how long these responses continue in the immunised host".

2. The response to the request from both reviewers to demonstrate internalised complexes with co-staining of intracellular compartments is not satisfactory. The authors argue that there are too few cells and this is why they cannot do this analysis. This is not the case as they have enough cells to visualise the antigen and the co-staining would be performed with the SAME cells.

3. Both reviewers have raised issues regarding the lack of statistical analysis. The argument that this cannot be performed due to interexperiment variation is invalid. The data across experiments can be normalised and then pooled.

We would request that the authors attempt to address these three points. The last point regarding statistics might be addressed by pooling data. The second point seems reasonable also, and some attempts should be made. The first point could be attempted, but the authors could provide a rationale if they strongly feel that the results of this experiment are not pertinent to the main point of the study, but if such is the case the authors should write an explanation why.

The journal does not encourage multiple rounds of revision and you should fully address the concerns of the referee in this final round of revision. Should you disagree with any of the refereesâ€™ concerns,

you should address this in your point-by-point response and provide solid scientific reasons for why you will not make the requested changes.

You should also pay close attention to the editorial comments included below. **In particular, please edit your figure legends to follow Journal standards as outlined in the editorial comments. Gating strategy as well as flurochrome axis labeling, and the percentage of cells/event count needs to be shown for all flow cytometry dot plots/histograms. Failure to do this will result in delays in the re-review process.**

Please note that submitting a revision of your manuscript does not guarantee eventual acceptance, and that your revision will be re-reviewed by the referees before a decision is rendered.

If the revision of the paper is expected to take more than three months, please inform the editorial office. Revisions taking longer than six months may be assessed by new referees to ensure the relevance and timeliness of the data.

Once again, thank you for submitting your manuscript to European Journal of Immunology and we look forward to receiving your revision.

Yours sincerely,
Nadja Bakocevic

On behalf of
Prof. Kenneth Murphy

Dr. Nadja Bakocevic
Editorial Office
European Journal of Immunology
e-mail: ejied@wiley.com

www.eji-journal.eu

Get the latest articles delivered directly to your desktop ! Register now for the free Wiley Alerting Service at
http://onlinelibrary.wiley.com/myprofile/alertManager

****

European Journal of Immunology

Reviewer: 1

Comments to the Author
In the revised version of the manuscript, the authors have adequately addressed reviewers' questions.

Reviewer: 2

Comments to the Author
The authors have not satisfactorily addressed my previous comments.

1. This comment has been ignored:
"The authors should determine the longevity of the CD8 and CD4 T cell division and CTL killing in response to IC-OVA to determine when these responses recede to undetectable levels. While it appears that CD4 T cell division is undetectable after one week, the CD8 T cell responses are still detectable. It would be interesting to know for how long these responses continue in the immunised host".

2. The response to the request from both reviewers to demonstrate internalised complexes with co-staining of intracellular compartments is not satisfactory. The authors argue that there are too few cells and this is why they cannot do this analysis. This is not the case as they have enough cells to visualise the antigen and the co-staining would be performed with the SAME cells.

3. Both reviewers have raised issues regarding the lack of statistical analysis. The argument that this cannot be performed due to interexperiment variation is invalid. The data across experiments can be normalised and then pooled.

Second Revision – authors' response
01-Feb-2018

Reviewer: 2

1. This comment has been ignored:
"The authors should determine the longevity of the CD8 and CD4 T cell division and CTL killing in response to IC-OVA to determine when these responses recede to undetectable levels. While it appears that CD4 T cell division is undetectable after one week, the CD8 T cell responses are still detectable. It would be interesting to know for how long these responses continue in the immunised host".

- We apologize that we didn't give an appropriate response to the suggested comment. It is indeed interesting to follow the CD8 and CD4 T cell division to know for how long an immunized host can induce responses. However, for the current study, this is not our main focus since our aim is to show the antigen presentation capacity of individual APC subsets in time as reflected in our title. We have previously shown that antigen presentation after administration of preformed immune complexes on DC can be detected up to 14 day by in vivo CD8 T cell proliferation (Van Montfoort et al. PNAS 2009). In the current study we show prolonged antigen presentation in vivo to transferred OVA-specific CD8 and CD4 T cells up to 1 week which is, in our opinion, sufficient to demonstrate the ability of splenic DC to engulf circulating in vivo formed antibody-antigen complexes and their capacity to sustain antigen presentation to CD8 and CD4 T cells. It would be interesting to follow up later time points but it is clear from figure 1 that after one week the antigen presentation capacity is already reduced to lower levels. Follow-up to later time points would not add more insight to our study.

2. The response to the request from both reviewers to demonstrate internalized complexes with co-staining of intracellular compartments is not satisfactory. The authors argue that there are too few cells and this is why they cannot do this analysis. This is not the case as they have enough cells to visualize the antigen and the co-staining would be performed with the SAME cells.

- Unfortunately the harvest amount of each individual subset which is antigen positive after sorting is very low (+/- 1000 cells). Analyzing the uptake of 1000 freshly sorted cells immediately after the sort with confocal microscopy is still achievable. However, doing co-stainings with antibodies and thereby extensively fixing, staining and washing the cells, is not doable with these low amounts of cells. However, we performed new experiments using lysotracker marker, which can be added directly to live cells to stain endo-lysosomal compartments without the risk of losing too many cells during the process. We have analyzed the cells and found co-localization between OVA antigen and lysotracker. Since this give some additional information about the intracellular localization of the antigen, we decided to add these data as Supplemental Figure 4 in the manuscript.

3. Both reviewers have raised issues regarding the lack of statistical analysis. The argument that this cannot be performed due to interexperiment variation is invalid. The data across experiments can be normalised and then pooled.

European Journal of Immunology

- Although normalization of all flow cytometry data is difficult, we have now added statistical analysis of average data of three pooled experiments as an additional figure (Figure 5C), showing significant differences between APC subsets in antigen presentation to CD8 or CD4 T cells. This convincingly shows differential MHCI and MHCII antigen presentation of the APC subsets at three different time points after in vivo administration (24, 48 and 72 hrs).

Third Editorial Decision
27-Mar-2018

Dear Dr. Ossendorp,

It is a pleasure to provisionally accept your manuscript entitled "Sustained cross-presentation capacity of splenic dendritic cell subsets in vivo" for publication in the European Journal of Immunology. For final acceptance, please follow the instructions below and return the requested items as soon as possible as we cannot process your manuscript further until all items listed below are dealt with.

Please note that EJI articles are now published online a few days after final acceptance (see Accepted Articles: https://onlinelibrary.wiley.com/toc/15214141/0/ja). The files used for the Accepted Articles are the final files and information supplied by you in Manuscript Central. You should therefore check that all the information (including author names) is correct as changes will NOT be permitted until the proofs stage.

We look forward to hearing from you and thank you for submitting your manuscript to the European Journal of Immunology.

Yours sincerely,
Nadja Bakocevic

on behalf of
Prof. Kenneth Murphy

Dr. Nadja Bakocevic
Editorial Office
European Journal of Immunology
e-mail: ejied@wiley.com
www.eji-journal.eu
